# Interleukin-6 and microRNA profiles induced by oral bacteria in human atheroma derived and healthy smooth muscle cells

**DOI:** 10.1186/s40064-015-0993-8

**Published:** 2015-04-30

**Authors:** Tanja Pessi, Leena E Viiri, Emma Raitoharju, Nagora Astola, Ilkka Seppälä, Melanie Waldenberger, Kari Lounatmaa, Alun H Davies, Terho Lehtimäki, Pekka J Karhunen, Claudia Monaco

**Affiliations:** Pirkanmaa Hospital District, University of Tampere School of Medicine and Fimlab Laboratories Ltd, Tampere, Finland; Kennedy Institute of Rheumatology, University of Oxford, London, UK; Research Unit of Molecular Epidemiology, Helmholtz Zentrum, German Research Center for Environmental Health, Munich, Germany; Institute of Epidemiology II, Helmholtz Zentrum, German Research Center for Environmental Health, Munich, Germany; Lounatmaa Ltd, Helsinki, Finland; Medical School, Tampere University, Tampere, FIN-33014 Finland

**Keywords:** Atherosclerosis, Inflammation, Genome wide expression, microRNA, Oral bacteria, Smooth muscle cells

## Abstract

**Background:**

Atherosclerosis is an inflammatory disease with possible contributions from bacterial antigens. We aimed to investigate the role of oral bacteria as inducers of inflammatory cascades in smooth muscle cells from carotid endarterectomy patients (AthSMCs) and healthy controls (HSMCs).

**Findings:**

Inactivated *Streptococcus mitis, S. sanguinis, S. gorgonii, Aggregatibacter actinomycetemcomitans* and *Porphyromonas gingivalis* were used to stimulate inflammation in HSMCs and AthSMCs. Tumor necrosis factor-α (TNFα) was used as a positive control in all stimulations. Interleukin-6 (IL-6) levels were determined from cell culture supernatants and microRNA expression profiles from cells after 24 h of bacterial stimulation. Genome wide expression (GWE) analyses were performed after 5 h stimulation. All studied bacteria induced pro inflammatory IL-6 production in both SMCs. The most powerful inducer of IL-6 was *A. actinomycetemcomitans* (p < 0.001). Of the 84 studied miRNAs, expression of 9 miRNAs differed significantly (p ≤ 0.001) between HSMCs and AthSMCs stimulated with inactivated bacteria or TNFα. The data was divided into two groups: high IL-6 producers (*A. actinomytectemcomititans* and TNFα) and low IL-6 producers (streptococcal strains and *P. gingivalis*). The expression of 4 miRNAs (miR-181-5p, −186-5p, −28-5p and −155-5p) differed statistically significantly (p < 0.001) between healthy HSMCs and AthSMCs in the low IL-6 producer group. According to multidimensional scaling, two gene expression clusters were seen: one in HSMCs and one AthSMCs.

**Conclusions:**

Our results suggest that inactivated oral bacteria induce inflammation that is differently regulated in healthy and atherosclerotic SMCs.

**Electronic supplementary material:**

The online version of this article (doi:10.1186/s40064-015-0993-8) contains supplementary material, which is available to authorized users.

## Introduction

Vascular smooth muscle cells (VSMCs) are highly differentiated muscle cells that form the medial layer of the vessel wall and control blood pressure by contracting and relaxing the vessels. SMCs play an important role in atherogenesis, including a key role in remodeling and plaque stabilization (Doran et al. 2008).

Multiple microRNAs (miRNAs), small non-protein-coding RNAs, are known to control the different phases of atherogenesis (reviewed in (Nazari-Jahantigh et al. 2012)). They are responsible for VSMC differentiation and proliferation under physiological or pathological conditions as well as modulating both adaptive and innate immune responses within SMC by encompassing every step from plaque formation to destabilization and rupture (Kang and Hata 2012). Although many miRNAs have been linked to atherosclerosis, only a few miRNAs (*e.g.* miR-21, −146a, −150, −155) have repeatedly been reported (Ma et al. 2013; Raitoharju et al. 2013).

Epidemiological studies have demonstrated a strong relationship between atherosclerosis and oral infections (Desvarieux et al. 2005; Desvarieux et al. 2013), possible due to indirect effects mediated through elevated pro inflammatory cytokines and other acute phase proteins. Several previous studies suggest however that oral bacteria can directly penetrate gingival tissues, enter the bloodstream and potentially induce transient bacteremia even after flossing, mastication, and tooth brushing (Li et al. 2000). Of oral pathogens, streptococci and periodontal bacteria are most frequently detected in atherosclerotic samples (Pessi et al. 2013). It is also known that oral pathogens such as *Porphyromonas gingivalis* and streptococci have the ability to invade human heart endothelial cells *in vitro* (Deshpande et al. 1998; Nagata et al. 2011) as well as accelerate plaque growth and macrophage invasion (Kesavalu et al. 2012). In our study, we evaluated the role of processed periodontal and endodontic bacteria in atherosclerotic inflammation using SMCs and selected bacterial species found in coronary plaques after heat inactivation (Pessi et al. 2013). Atherosclerotic inflammation was studied by measuring pro inflammatory interleukin-6 (IL-6) levels and gene expression profiles after bacterial stimulation.

## Methods

### Bacterial strains

*Streptococcus mitis* ATCC 49456, *Streptococcus sanguinis* ATCC 10556, *Streptococcus gorgonii* ATCC 10558, *Aggregatibacter actinomycetemcomitans* ATCC 700685, *Porphyromonas gingivalis* ATCC 33277 from stock culture collection (LCG Standards AB, Borås, Sweden) were diluted to 10^8^/ml in sterile phosphate buffered saline (PBS). All bacteria were heat-inactivated and filtered through a 0.45 μm pore size filter (Merck Millipore, Darmstadt, Germany) before being added into cell cultures as previously described (Pessi et al. 1999). These bacterial preparations were stored at −80°C prior to cell culture experiments.

### *Ex vivo* culture of cells isolated from human atherosclerotic plaques and healthy donors

Smooth muscle cells (SMC) were isolated from the carotid endarterectomies of 4 patients undergoing revascularization procedures for symptomatic carotid disease at Charing Cross Hospital, London. SMCs were isolated and cultures produced as in Monaco et al. 2004. Aortic SMCs from 2 healthy donors (HSMCs) were purchased from PromoCell Ltd (Heidelberg, Germany). The study was approved by the Research Ethics Committee (Riverside Research Ethics Committee, London). All patients gave written informed consent according to the Human Tissue Act 2004 (UK).

SMCs were grown in SMC growth medium 2 (PromoCell Ltd, Heidelberg, Germany). Viability was monitored with the use of 3-(4,5-dimethyl-2-yl)-2,5-diphenyltetrazolium (MTT) (Sigma-Aldrich, Carlsbad, California, USA). After 3–4 passages, SMCs were cultured in Smooth Muscle Cell Growth Medium 2 (PromoCell) either alone or in the presence of each of the bacterial preparations separately at a final concentration of 10^7^, 10^6^ and 10^5^/ml of culture media. Tumor necrosis factor alpha (TNF-α) (InvivoGen, Source BioScience LifeSciences, Nottingham, UK) was used as a positive control at a concentration of 10 ng/ml. After culturing SMCs with or without bacterial stimulation for 24 h, cell culture supernatants were collected for IL-6 measurement, and cell pellets for miRNA expression profiling. Separate experiments were performed for GWE analyses using the same bacterial preparations. Pellets for GWE analyses were collected after 5 h of stimulation.

### Interleukin-6 measurements

Cell culture supernatants were removed after 24 hours and stored at −80°C for single-batch cytokine analysis using the DuoSet ELISA Development Systems (R&D Systems, Minneapolis, USA), according to the manufacturer’s instructions. The ELISA detection limit was 2 pg/ml. Experiments were performed in triplicate.

### RNA isolation and expression profiling

Total RNA for miRNA and GWE was isolated using the miRNeasy Mini Kit (Qiagen Ltd, Valencia, USA), and miRNA profiling of 84 commonly known miRNAs was performed using miScript miRNA Array (MIHS-001Z, Qiagen) according to the manufacturer’s instructions. Due to its lowest standard deviation between runs, SNORD68 was selected as a housekeeping gene. Samples with a Ct value ≥35 were excluded from analyses. Of 2016 measurements, 17 failures were observed and excluded from further analyses.

Whole genome gene expression (GWE) analysis was performed with Illumina DirectHyb HumanHT-12 v4.0 (Illumina, Inc., San Diego, USA) containing 47,231 oligonucleotide probes representing 34,602 genes, and processed according to the manufacturer’s protocol. The normalization was applied by subtracting the signal intensity of each probe in each sample by the mean intensity for that sample across all probes (global mean normalization). After normalization, multidimensional scaling (MDS) was used to illustrate global gene expressions of each sample in two-dimensional spaces (Cox and Cox 2001).

### Transmission electron microscopy

**Figure 1:**
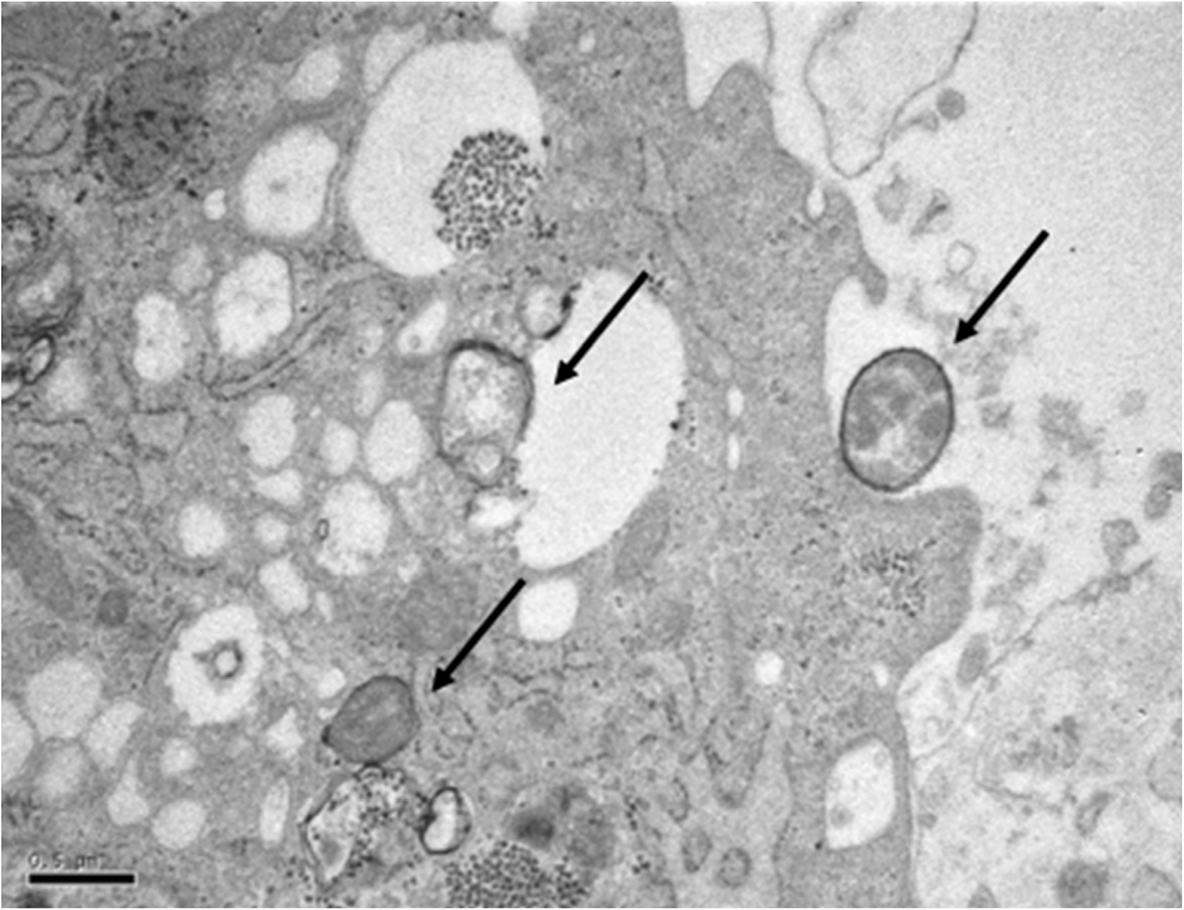
Phagocytosis of *S. mitis* by AthSMCs. Arrows indicate bacteria and their components. Bar is 0.5 μm.

To visualize the bacterial processing in SMCs, AthSMCs were selected after 24 h stimulation with *S. mitis* for electron microscopy (EM, Figure 1) with a JEOL 1200EX transmission electron microscope (Japanese Electron Optics Laboratory, Tokyo, Japan) operating at 60 kV.

### Statistical analyses

All results (IL-6 levels, miRNA and GWE) were normalized against the corresponding results obtained from cultures without the addition of inactivated bacteria. Due to the skewed distributions and low number of cases, statistical differences between the experiments were calculated using the non-parametric Mann Whitney *U* test and Spearman’s correlation test. Of IL-6 results p < 0.01 was considered to be statistically significant, and due to the multiple measurements of the miRNA results p ≤ 0.001 was considered statistically significant (PASW Statistical Software version 18; SPSS Ltd, Quarry Bay, Hong Kong, China).

## Results

### IL-6 production induced by oral bacteria

**Figure 2:**
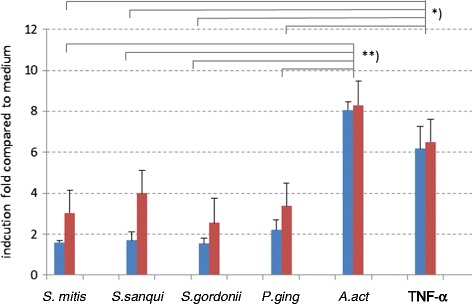
The production of IL-6 induced by inactivated oral bacteria at the concentration of 10^7^/ml and TNF-α (10 ng/ml) in healthy SMCs (HSMCs, n = 2) and atheroma derived SMCs (AthSMCs, n = 4). Means (+SD) are expressed as induction folds compared to medium values (no stimulus). Statistically significant differences (*; p = 0.01 and **; p = 0.001) are marked in the figure. *S. Streptococcus*; *P. ging*, *Porphyromonas gingivalis*; *A. act, Aggregatibacter actinomycetemcomitans;* TNF*-*α*,* tumor necrosis factor*-*α*.* Red bar represents from AthSMCs results and blue from HSMCs.

All studied bacteria (S*. mitis, S. sangui, S. gordonii, P. gingivalis, A. actinomycetemcomitans)* and TNFα induced IL-6 production in AthSMCs as well as in healthy SMCs (Figure 2). Among bacterial stimulations, the most powerful inducer was *A. actinomycetemcomitans* (p < 0.001, Mann Whitney *U* test). IL-6 responses induced by other bacteria did not differ from each other (p>0.01). The effects of bacteria on IL-6 production were dose-dependent in both cell types (data not shown).

### miRNA and GWE profiles in SMCs after stimulation

**Figure 3:**
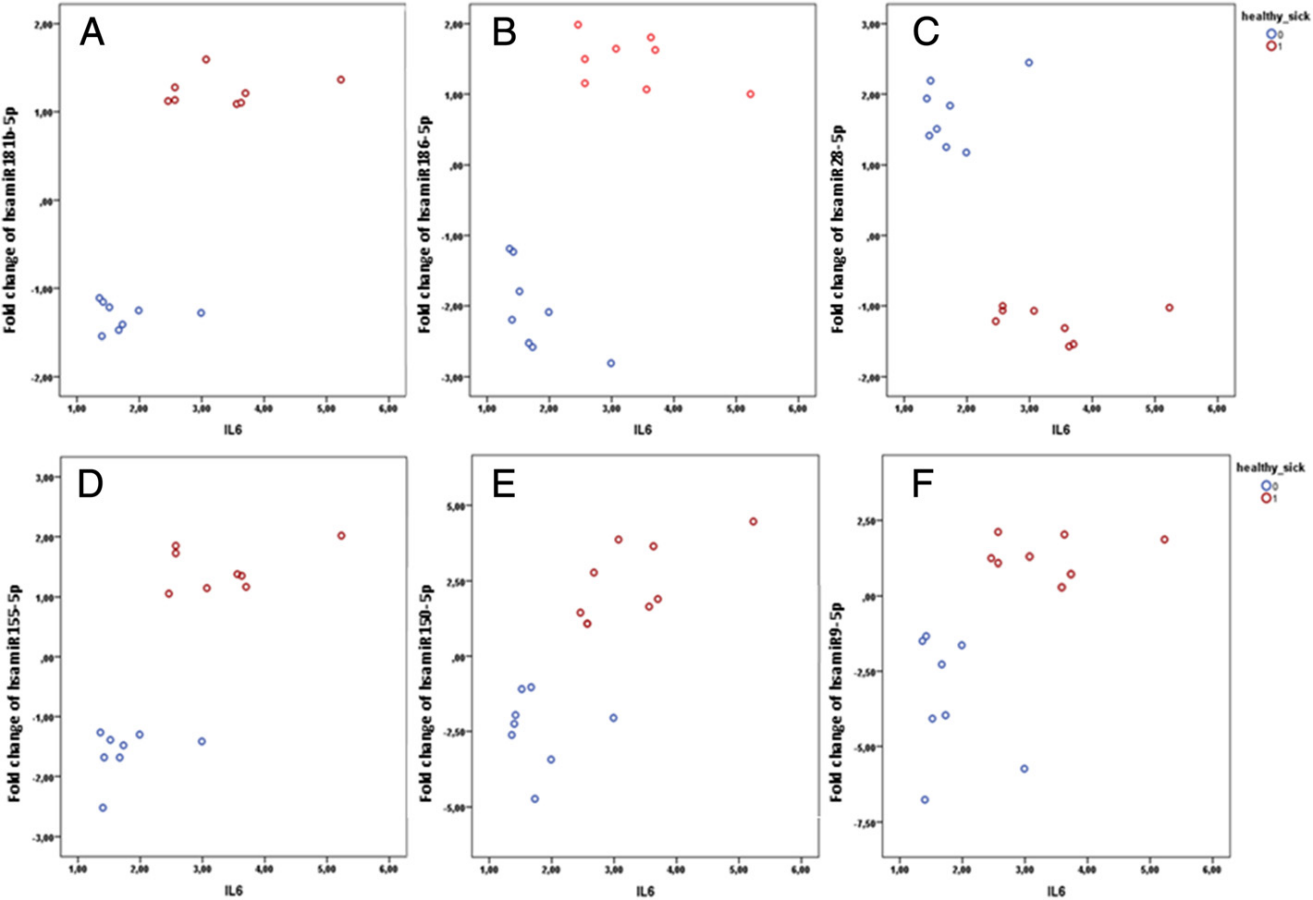
Fold changes of miRNAs (**A**; miR-181b-5p, **B**;186-5p, **C**; 28-5p, **D**;155-5p, **E**;150-5p, **F**; 9-5p) and IL-6 levels after streptococci or *P. gingivalis* stimulation in healthy SMCs (HSMCs) and atheroma derived SMCs (AthSMCs). N-fold difference compared to corresponding values from cultures without bacteria. Each stimulation was performed twice, red circles represent AthSMCs results and blue circles from HSMCs.

miRNA expression profiles in AthSMCs and HSMCs were studied after 24 h bacterial stimulation. Of the 84 miRNAs, 9 (miR-96-5p, −185-5p, −181b-5p, −200c-3p, −28-5p, −222-3p, −186-5p, let7a-5p and let7e-5p) were statistically significantly differently expressed (Table 1 and Additional file 1: Table S1) in AthSMC and HSMCs after bacterial stimulation (p < 0.001). Since IL-6 levels produced by SMCs after stimulation with *A. actinomytectemcomititans* significantly differed to IL-6 levels after stimulation with other bacteria, the data was divided into two groups: “high IL-6 producers” *i.e. A. actinomytectemcomititans* and TNF-α and “low IL-6 producers”, *i.e*. different streptococci and *P. gingivalis*. It was observed that in the “low IL-6 producer” group (Figure 3), the expression of 4 miRNAs (miR-181b-5p, −186-5p, −28-5p and −155-5p) differed statistically significantly between HSMCs and AthSMCs (p <0.001; Mann- Whitney *U* test). The expression of 3 miRNAs (miR-155-5p, −150-5p and −9-5p) correlated with IL-6 levels (p < 0.001, Spearman’s correlation).

**Table 1:** **Fold changes of stimulated miRNAs in healthy and atherosclerotic SMCs**

		**HSMCs**			**AthSMCs**			
			**Percentiles**			**Percentiles**		
**miRNA ID**	**Accession number**	**Median**	**25th**	**75th**	**Median**	**25th**	**75th**	p-value*
hsa-miR-142-5p	MIMAT0000433	−1.58	−1.98	0.67	−0.05	−1.89	2.08	
hsa-miR-9-5p	MIMAT0000441	−2.31	−4.05	−1.49	−1.51	−2.33	1.71	0.045
hsa-miR-150-5p	MIMAT0000451	−2.01	−2.69	−1.03	3.18	−0.4	4.31	0.002
hsa-miR-27b-3p	MIMAT0000419	−1.25	−1.53	1.05	−1.42	−1.74	0.68	
hsa-miR-101-3p	MIMAT0000099	3.39	1.61	5.33	1.05	−1.47	2.05	0.007
hsa-let-7d-5p	MIMAT0000065	−1.26	−1.47	−1.05	1.05	−1.07	1.1	0.014
hsa-miR-103a-3p	MIMAT0000101	−0.02	−1.32	1.44	−1.07	−1.28	1.1	
hsa-miR-16-5p	MIMAT0000069	−1.32	−1.76	0.48	−0.02	−1.17	1.19	
hsa-miR-26a-5p	MIMAT0000082	−1.18	−1.4	1.2	−1.78	−2.04	−1.15	
hsa-miR-32-5p	MIMAT0000090	1.22	−1.12	3.4	4.01	1.56	5.69	
hsa-miR-26b-5p	MIMAT0000083	−1.37	−1.87	−1.08	−1.77	−2.19	−1.32	
hsa-let-7 g-5p	MIMAT0000414	−1.41	−1.88	−1.18	−1.23	−1.56	0.53	
hsa-miR-30c-5p	MIMAT0000244	−1.19	−1.52	1.09	−1.71	−2.25	−1.11	
**hsa-miR-96-5p**	**MIMAT0000095**	**−3.54**	**−4.93**	**−1.53**	**1.43**	**1.13**	**3.41**	**<0.001**
**hsa-miR-185-5p**	**MIMAT0000455**	**2.07**	**1.43**	**3.15**	**−1.25**	**−1.3**	**−1.02**	**<0.001**
hsa-miR-142-3p	MIMAT0000434	−1.11	−1.54	2.65	2.18	1.9	3.98	0.014
hsa-miR-24-3p	MIMAT0000080	1.03	−1.13	1.25	−1.12	−1.48	1.22	
hsa-miR-155-5p	MIMAT0000646	−1.4	−1.63	−1.18	1.06	−1.12	1.31	0.003**
hsa-miR-146a-5p	MIMAT0000449	−1.61	−2.01	−1.17	−2.01	−2.48	−1.32	
hsa-miR-425-5p	MIMAT0003393	1.24	−0.5	1.49	−1.16	−1.65	0.61	
**hsa-miR-181b-5p**	**MIMAT0000257**	**−1.27**	**−1.39**	**−1.14**	**1.13**	**−0.55**	**1.34**	<0.001**
hsa-miR-302b-3p	MIMAT0000715	−1.97	−3.13	0.46	−1.84	−2.83	−1.15	
hsa-miR-30b-5p	MIMAT0000420	−1.09	−1.3	1.13	−1.25	−1.54	0.6	
hsa-miR-21-5p	MIMAT0000076	−1.69	−2.72	−1.37	−1.98	−2.36	−1.23	
hsa-miR-30e-5p	MIMAT0000692	1.46	1.09	2.09	−1.15	−1.45	1.18	0.008
**hsa-miR-200c-3p**	**MIMAT0000617**	**−1.79**	**−2.51**	**−1.5**	**1.47**	**1.04**	**2.38**	**<0.001**
hsa-miR-15b-5p	MIMAT0000417	−1.06	−1.29	1.12	−0.09	−1.47	1.24	
hsa-miR-223-3p	MIMAT0000280	−2.6	−5.45	−1.14	−1.48	−1.8	7.44	
hsa-miR-194-5p	MIMAT0000460	1.06	−1.24	1.47	−1.65	−2.01	0.59	
hsa-miR-210-3p	MIMAT0000267	2.65	1.39	4.49	2.02	1.52	2.34	
hsa-miR-15a-5p	MIMAT0000068	2.07	1.61	2.95	1.14	−1.4	1.8	0.024
hsa-miR-181a-5p	MIMAT0000068	1.78	1.33	2.72	1.34	−1.29	1.58	0.028
hsa-miR-125b-5p	MIMAT0000423	1.1	−1.11	1.5	−1.51	−2.11	−1.16	0.01
hsa-miR-99a-5p	MIMAT0000097	1.07	−1.12	1.41	−1.74	−2.04	0.55	
**hsa-miR-28-5p**	**MIMAT0000085**	**1.82**	**1.3**	**2.13**	**−1.43**	**−1.78**	**1.05**	<0.001**
hsa-miR-320a	MIMAT0000510	−0.02	−1.46	1.22	1.13	−1.05	1.38	
hsa-miR-125a-5p	MIMAT0000443	−1.48	−3.1	0.55	−1.21	−1.89	1.42	
hsa-miR-29b-3p	MIMAT0000100	2.02	1.39	4.66	2	−1.1	2.65	
hsa-miR-29a-3p	MIMAT0000086	1.43	−0.57	1.86	−1.03	−1.18	1.57	
hsa-miR-141-3p	MIMAT0000432	−0.09	−1.98	2.1	2.08	−0.49	3.03	
hsa-miR-19a-3p	MIMAT0000073	1.82	−0.51	3.2	−1.06	−1.37	1.69	
hsa-miR-18a-5p	MIMAT0000072	0.05	−1.19	1.6	−0.04	−1.28	1.13	
hsa-miR-374a-5p	MIMAT0000727	−1.28	−1.56	0.5	−1.53	−2.1	−1.03	
hsa-miR-423-5p	MIMAT0004748	−1.2	−1.34	0.5	1.2	1.02	1.52	0.002
**hsa-let-7a-5p**	**MIMAT0000062**	**−1.53**	**−1.9**	**−1.26**	**−1.02**	**−1.13**	**1.13**	**0.001**
hsa-miR-124-3p	MIMAT0000422	1.15	−1.86	3.06	1.93	1.13	3.97	
hsa-miR-92a-3p	MIMAT0000092	−0.03	−1.37	1.22	−1.06	−1.13	1.18	
hsa-miR-23a-3p	MIMAT0000078	−1.34	−1.98	−1.04	−1.5	−1.7	−1.13	
hsa-miR-25-3p	MIMAT0000081	−1.47	−2.14	−1.27	−1.73	−1.97	−1.08	
**hsa-let-7e-5p**	**MIMAT0000066**	**−1.55**	**−1.87**	**−1.26**	**1.16**	**−1.04**	**1.32**	**<0.001**
hsa-miR-376c-3p	MIMAT0000720	2.08	1.34	2.73	−1.43	−1.63	1.5	0.006
hsa-miR-126-3p	MIMAT0000445	1.2	−1.08	1.98	−0.09	−1.52	1.44	
hsa-miR-144-3p	MIMAT0000436	−2.07	−3.78	1.88	1.21	−1.49	3.64	
hsa-miR-424-5p	MIMAT0001341	−1.46	−2.18	−1.09	−1.63	−1.78	0.44	
hsa-miR-30a-5p	MIMAT0000087	1.6	1.1	2.06	−0.03	−1.24	1.35	
hsa-miR-23b-3p	MIMAT0000418	−1.5	−1.99	0.48	−1.24	−1.61	0.5	
hsa-miR-151a-5p	MIMAT0004697	−0.01	−1.21	1.35	−1.59	−2.01	−1.13	0.02
hsa-miR-195-5p	MIMAT0000461	−1.07	−1.27	0.53	1.22	1.13	1.45	0.005
hsa-miR-143-3p	MIMAT0000435	2.26	1.85	4.34	1.28	−0.5	2.15	
hsa-miR-30d-5p	MIMAT0000245	0.02	−1.48	1.42	−1.14	−1.38	1.15	
hsa-miR-191-5p	MIMAT0000440	−1.1	−1.24	1.23	−1.47	−1.73	0.53	
hsa-let-7i-5p	MIMAT0000415	1.15	1.08	1.41	1.1	−1.16	1.32	
hsa-miR-302a-3p	MIMAT0000684	−1.06	−1.19	1.4	−1.25	−1.85	1.13	
**hsa-miR-222-3p**	**MIMAT0000279**	**1.4**	**−0.53**	**1.85**	**−1.52**	**−1.65**	**0.55**	**<0.001**
hsa-let-7b-5p	MIMAT0000063	1.14	−1.09	1.42	1.96	1.53	2.14	0.002
hsa-miR-19b-3p	MIMAT0000074	1.64	−1.4	3.14	0.07	−1.15	1.67	
hsa-miR-17-5p	MIMAT0000070	1.63	1.26	2.28	−1.02	−1.32	1.3	0.002
hsa-miR-93-5p	MIMAT0000093	1.23	−1.18	2.45	0.01	−1.45	1.35	
**hsa-miR-186-5p**	**MIMAT0000456**	**−1.94**	**−2.44**	**−1.37**	**1.34**	**−0.54**	**1.64**	<0.001**
hsa-miR-196b-5p	MIMAT0001080	1.46	−3.14	7.09	1.81	−1.24	6.42	
hsa-miR-27a-3p	MIMAT0000084	1.33	−0.54	1.65	−1.04	−1.34	1.36	
hsa-miR-22-3p	MIMAT0000077	2.42	1.44	4.24	1.35	−0.6	2.19	0.003
hsa-miR-130a-3p	MIMAT0000425	1.6	1.15	2.06	2.05	−0.6	3.12	
hsa-let-7c	MI0000064	−1.2	−1.35	1.17	0.01	−1.08	1.57	
hsa-miR-29c-3p	MIMAT0000681	1.41	−0.53	1.84	1.04	−1.34	1.25	
hsa-miR-140-3p	MIMAT0004597	1.69	−0.57	3.06	−1.24	−1.62	1.1	0.008
hsa-miR-128-3p	MIMAT0000424	−1.18	−1.48	0.58	−1.44	−1.73	0.48	
hsa-let-7f-5p	MIMAT0000067	−1.6	−1.91	−1.39	−1.4	−2.02	−1.14	
hsa-miR-122-5p	MIMAT0000421	−1.84	−4.05	−1.01	1.65	−0.59	3.24	0.028
hsa-miR-20a-5p	MIMAT0000075	1.36	−0.53	2	−1.38	−1.56	0.53	0.004
hsa-miR-106b-5p	MIMAT0000680	2.06	−0.41	2.69	−0.03	−1.27	1.23	0.006
hsa-miR-7-5p	MIMAT0000252	−1.53	−2.98	−1.37	−1.45	−2.61	0.49	
hsa-miR-100-5p	MIMAT0000098	−1.05	−1.36	1.19	−1.85	−2.09	1.05	
hsa-miR-302c-3p	MIMAT0000717	−1.17	−1.74	3.62	1.9	1.2	2.27	

*Mann–Whitney *U* test.

**When data was divided into low and high IL-6 stimulants, highly significant results (P < 0.001) were observed in the group of low IL-6 stimulants, like streptococci and *P. gingivalis* (see Figures 2 and 3).

Fold changes calculated using unstimulated cells as a reference. The data from different stimuli (streptococci, *P. gingivalis*, *A. actinomytectemcomititans* or TNFα) were pooled and median values presented with 25^th^ and 75^th^ interquartile ranges. P-values < 0.05 are presented. P-values < 0.001 in bold.

**Figure 4:**
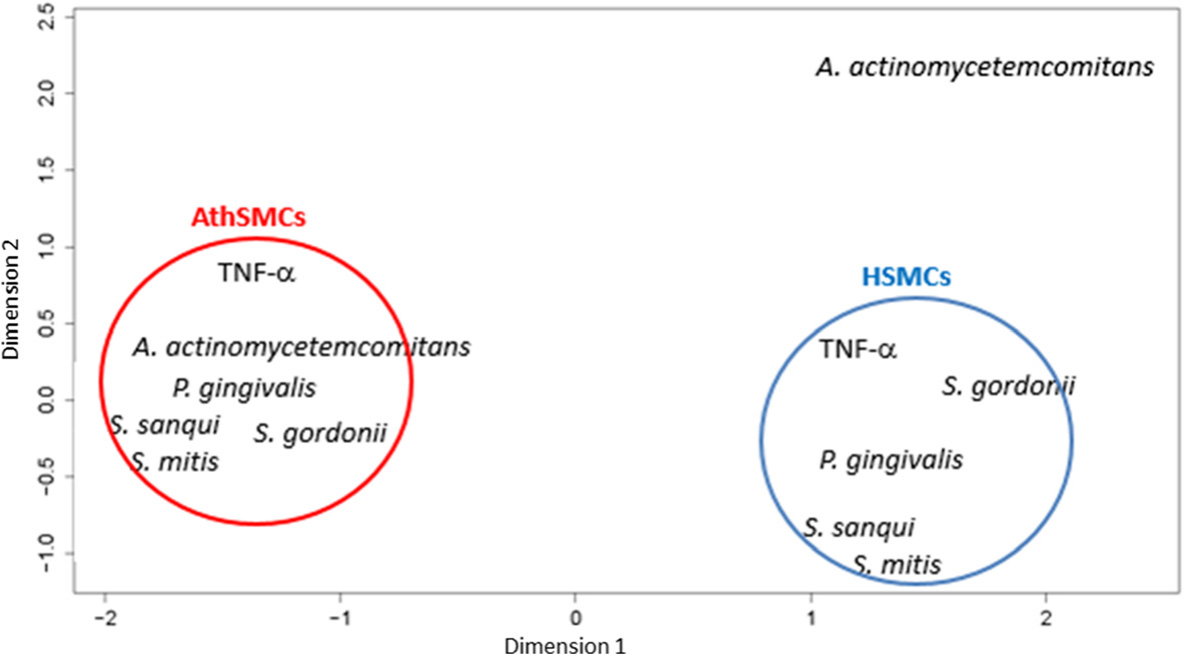
Dimensions 1 and 2 from the multidimensional scaling (MDS) of GWE data in healthy SMCs (HSMCs) and atheroma derived SMCs (AthSMCs) stimulated with oral bacteria and tumor necrosis factor (TNF)*-*α, *S., Streptococcus*; *P., Porphyromonas*; *A., Aggregatibacter.* Red circles represent AthSMCs results and blue from HSMCs.

Multidimensional scaling (MDS) from GWE data was performed for illustrative purposes to evaluate global expression of stimulated genes in SMCs (Figure 4). Global gene expression profiles were similar for samples stimulated with oral bacteria within the same SMC type with the exception of by *A. actinomycetemcomitans* in HSMCs. Two expression profile cluster were observed according to cell type: HSMC and AthSMC. This difference was clearly seen in dimension 1 vs. 2 (Figure 4) as well as in dimension 1 vs. 3 (data not shown).

## Discussion and conclusion

The present results confirm that the studied attenuated oral bacteria have the ability to activate a pro-inflammatory response in vascular smooth muscle cells. *A. actinomycetemcomitans* was the most potent inducer of inflammation and differed from other bacteria in its capacity to induce inflammation. This was seen in both IL-6 levels and gene expression profiles.

After bacterial stimulation miRNA profiles were different in atherosclerotic SMCs and in healthy SMCs, which has not been studied before. The down regulation of miR-185-5p after bacterial stimulation in AthSMCs was in line with studies on carotid plaques without bacterial stimulation (Raitoharju et al. 2011; Raitoharju et al. 2013) suggesting that bacterial stimulation *per se* does not change the direction of miRNA expression. Expression levels (with or without bacterial stimulation) of other 8 miRNAs (miR-96-5p, −181b-5p, −185-5p, −200c-3p, −222-3p, −28-5p, let-7a-5p) in atherosclerotic tissues have not been identified before (see supplement summary), however their characterized functions (explained in the Additional file 1: Table S1) support their involvement in atherosclerotic inflammation.

miRNA results from ‘the low-IL-6 producers’, *i.e.* stimulation with streptococci and *P. gingivalis* were separately evaluated. Our result show down-regulation of miR 28-5p, which may result in the enhancement of several inflammatory markers and adhesion molecules, as suggested by Stather et al. (2013). We also detected upregulated miR-181b-5p and −186-5p, which may suppress plasminogen activator inhibitor-1 in VSMCs (Chen et al. 2014) and promote apoptosis (Zhou et al. 2008), thus interfering with extracellular matrix degradation, and structural and functional changes in VSMCs. miR-155 is highly expressed in various cell types including VSMCs and endothelial cells (Faraoni et al. 2009). It changes endothelial cell morphology and modulates the endothelial phenotype via re-organizing the actin cytoskeleton (Weber et al. 2014). Upregulated miR-155 also attenuates endothelial cell migration, proliferation, and apoptosis in atherosclerotic plaques (Weber et al. 2014). miR-155-5p was highly expressed in AthSMCs after bacterial stimulation and its expression also correlated with IL-6 levels, suggesting a potential role for this miRNA in bacterial-induced atherosclerotic inflammation.

Multidimensional scaling (MDS) summarizes genome wide expression (GWE) data of each sample and displays a structure of distance-like data as a geometrical picture illustrating similarities or dissimilarities between each sample (Cox and Cox 2001). Dots in the picture of dimensions 1 vs. 2 demonstrate major variances occurring in global gene expressions. Dots in MDS figures do not, however, explain which and how many genes are differently expressed. Two major clusters were seen here, *i.e.* one among HSMCs and one among AthSMCs. This is in line with our findings observed in the miRNA profiles.

Although our study is a pilot study with limited numbers, it is a novel study looking at the effects of attenuated oral bacteria in inducing inflammation in both healthy and atherosclerotic SMCs. No previous studies have combined both traditional inflammatory markers, like IL-6, as well as newly discovered gene expression markers in bacterial-induced atherosclerosis. The relatively low expression levels induced by attenuated bacteria may indicate that certain bacterial antigens from attenuated bacterial cells could have some role in smouldering atherosclerotic inflammation.

## Additional file

Additional file 1: Table S1. Function of 9 miRNAs differently expressed in AthSMC and HSMCs after streptococci and *P. gingivalis* stimulation.

## Abbreviations

AthSMCs: Smooth muscle cells from carotid endarterectomy patients
EM: Electron microscopy
GWE: Genome wide expression
HSMC: Smooth muscle cells from healthy donors
IL: Interleukin
MDS: Multi-dimensional scaling
miRNA: MicroRNA
P: *Porphyromonas*
SMCs: Smooth muscle cells
TNF: Tumor necrosis factor
VSMCs: Vascular smooth muscle cells
