# Supplementary material for: Interleukin-6 and microRNA profiles induced by oral bacteria in human atheroma derived and healthy smooth muscle cells
Source: Springerplus. 2015 Apr 30;4:206. doi: 10.1186/s40064-015-0993-8 (PMC4424225; doi:10.1186/s40064-015-0993-8)
Supplement: Additional file 1: Table S1. — Function of 9 miRNAs differently expressed in AthSMC and HSMCs after streptococci and P. gingivalis stimulation. [file 40064_2015_993_MOESM1_ESM.docx]

**Table 1S**. Function of 9 miRNAs differently expressed in AthSMC and HSMCs after streptococci and *P. gingivalis* stimulation.

|  | **Our result in AthSMCs** | **Function** | **Ref.** |
| --- | --- | --- | --- |
| miR-28-5p | down | down regulated in blood of peripheral arterial disease (PAD) and possible biomarker for PAD, expression in smooth muscle cell (SMC) or atherosclerotic tissues unidentified, enhances the expression of nuclear factor of kappa light polypeptide gene enhancer in B cells and adhesion molecules | Stather et al 2013 |
| miR-96-5p | up | upregulated in various cancers, inhibits specific gene expression in SMC, interferes with contraction of vascular SMC and suppresses the uptake high density lipoprotein cholesterol | Lin et al 2010,  Kim et al 2014,  Wang et al 2013 |
| miR-181b-5p | up | expression depends on cell types, inhibits the expression of plasminogen activator inhibitor-1 in vascular SMCs | Yang et al 2014,  Chen et al 2014 |
| miR-185-5p | down | down regulated in B cells and atherosclerotic/carotid plaques, regulates angiogenesis and downstream signaling of vascular endothelial growth factor receptor 2 | Belver et al 2010, Raitoharju et al 2011, Yuan et al 2014 |
| miR-186-5p | up | upregulated in epithelial cancer cells, expression in SMC or atherosclerotic tissues unidentified, induces apoptosis | Zhou et al 2008 |
| miR-200c-3p | up | upregulated in vascular SMCs and aortas in mice, regulator of cell differentiation, intracellular modulator by modifying efficiency of toll like receptor-4 signaling through the MyD88-dependent pathway, enhances inflammatory responses, *i.e.* cyclooxygenase-2 and monocyte chemoattractant protein-1, and promotes monocyte binding | Reddy et al 2012,  Wendlandt et al 2012 |
| miR-222-3p | down | up/down regulated in macrophages depending on stimulus, expression in SMC or atherosclerotic tissues unidentified, linked to vasculature development of | Graff et al 2012, Boettger et al 2012 |
|  |  |  |  |
| let-7a-5p | down | down regulated in blood of type 2 diabetic patients, highly expressed in endothelial cells, expression in SMC or atherosclerotic tissues unidentified, inhibits oxidized low density lipoprotein-induced epithelial cell injury | Santovito et al 2014, Bao et al 2014 |
| let-7e-5p | up | down regulated in blood of peripheral arterial disease, expression in SMC or atherosclerotic tissues unidentified, upregulates vascular endothelial growth factor and nuclear factor of kappa light polypeptide gene enhancer in B cells | Stather et al 2013,  Rao et al 2015 |

**References**

Bao MH, Zhang YW, Lou XY, Cheng Y, Zhou HH (2014) Protective effects of let-7a and let-7b on oxidized low-density lipoprotein induced endothelial cell injuries. PLoS One 9(9):e106540

Belver L, de Yébenes VG, Ramiro AR (2010) MicroRNAs prevent the generation of autoreactive antibodies. Immunity 33(5):713-722

Boettger T, Braun T (2012) A new level of complexity: the role of microRNAs in cardiovascular development. Circ Res 110(7):1000-1013

Chen YS, Shen L, Mai RQ, Wang Y (2014) Levels of microRNA-181b and plasminogen activator inhibitor-1 are associated with hypertensive disorders complicating pregnancy. Exp Ther Med 8(5):1523-1527

Graff JW, Dickson AM, Clay G, McCaffrey AP, Wilson ME (2012) Identifying functional microRNAs in macrophages with polarized phenotypes.J Biol Chem 287(26):21816-21825

Kim S, Hata A, Kang H (2014) Down-regulation of miR-96 by bone morphogenetic protein signaling is critical for vascular smooth muscle cell phenotype modulation. J Cell Biochem 115(5):889-95

Lin H, Dai T, Xiong H, Zhao X, Chen X, Yu C, Li J, Wang X, Song L (2010) Unregulated miR-96 induces cell proliferation in human breast cancer by downregulating transcriptional factor FOXO3a. PLoS One 5: e15797

Raitoharju E, Lyytikäinen LP, Levula M, Oksala N, Mennander A, Tarkka M, Klopp N, Illig T, Kähönen M, Karhunen PJ, Laaksonen R, Lehtimäki T (2011)miR-21, miR-210, miR-34a, and miR-146a/b are up-regulated in human atherosclerotic plaques in the Tampere Vascular Study.Atherosclerosis 219(1):211-217

Rao R, Nagarkatti P, Nagarkatti M (2015) Role of miRNA in the Regulation of Inflammatory Genes in Staphylococcal Enterotoxin B-Induced Acute Inflammatory Lung Injury and Mortality. Toxicol Sci. Jan 5. pii: kfu315

Reddy MA, Jin W, Villeneuve L, Wang M, Lanting L, et al. (2012) Pro-inflammatory role of microrna-200 in vascular smooth muscle cells from diabetic mice. Arterioscler Thromb Vasc Biol 32(3):721-729

Santovito D1, De Nardis V, Marcantonio P, Mandolini C, Paganelli C, Vitale E, Buttitta F, Bucci M, Mezzetti A, Consoli A, Cipollone F (2014). Plasma exosome microRNA profiling unravels a new potential modulator of adiponectin pathway in diabetes: effect of glycemic control.J Clin Endocrinol Metab. 99(9):E1681-1685

Stather PW1, Sylvius N, Wild JB, Choke E, Sayers RD, Bown MJ (2013). Differential microRNA expression profiles in peripheral arterial disease. Circ Cardiovasc Genet 6(5):490-497

Wang, L, Jia, XJ, Jiang HJ, DuY, Yang F, Si SY, Hong B (2013). MicroRNAs 185, 96, and 223 repress selective high-density lipoprotein cholesterol uptake through posttranscriptional inhibition. Molec Cell Biol 33(10): 1956-1964

Wendlandt EB1, Graff JW, Gioannini TL, McCaffrey AP, Wilson ME (2012).The role of microRNAs miR-200b and miR-200c in TLR4 signaling and NF-κB activation. Innate Immun 18(6):846-855

Yang L, Wang YL, Liu S, Zhang PP, Chen Z, Liu M, Tang H (2014) miR-181b promotes cell proliferation and reduces apoptosis by repressing the expression of adenylyl cyclase 9 (AC9) in cervical cancer cells. FEBS Lett 588(1):124-130

Yuan HX, Zhang JP, Kong WT, Liu YJ, Lin ZM, Wang WP, Guo JM (2014) Elevated microRNA-185 is associated with high vascular endothelial growth factor receptor 2 expression levels and high microvessel density in clear cell renal cell carcinoma. Tumour Biol 35(12):12757-12763

Zhou L, Qi X, Potashkin JA, Abdul-Karim FW and Gorodeski GI (2008) MiRNAs miR-186 and miR-150 down-regulate expression of the pro-apoptotic purinergic P2X7 receptor by activation of instability sites at the 3'-untranslated region of the gene that decrease steady-state levels of the transcript. J Biol Chem 83(42):28274-28286
